# Pulse pressure variation guided goal-direct fluid therapy decreases postoperative complications in elderly patients undergoing laparoscopic radical resection of colorectal cancer: a randomized controlled trial

**DOI:** 10.1007/s00384-024-04606-x

**Published:** 2024-03-04

**Authors:** Qiu-Rong Wu, Zi-Zuo Zhao, Ke-Ming Fan, Hui-Ting Cheng, Bin Wang

**Affiliations:** 1 Department of Anesthesiology, the First Affiliated Hospital of Chongqing Medical University, Chongqing, 400016 China; 2 Department of Anesthesiology, Yongchuan District People’s Hospital of Chongqing, Chongqing, 400016 China

**Keywords:** PPV, Goal-directed fluid therapy, Laparoscopic, Elderly, Radical resection of colorectal cancer, Complications

## Abstract

**Objective:**

The use of goal-directed fluid therapy (GDFT) has been shown to reduce complications and improve prognosis in high-risk abdominal surgery patients. However, the utilization of pulse pressure variation (PPV) guided GDFT in laparoscopic surgery remains a subject of debate. We hypothesized that utilizing PPV guidance for GDFT would optimize short-term prognosis in elderly patients undergoing laparoscopic radical resection for colorectal cancer compared to conventional fluid therapy.

**Methods:**

Elderly patients undergoing laparoscopic radical resection of colorectal cancer were randomized to receive either PPV guided GDFT or conventional fluid therapy and explore whether PPV guided GDFT can optimize the short-term prognosis of elderly patients undergoing laparoscopic radical resection of colorectal cancer compared with conventional fluid therapy.

**Results:**

The incidence of complications was significantly lower in the PPV group compared to the control group (32.8% vs. 57.1%, *P* = .009). Additionally, the PPV group had a lower occurrence of gastrointestinal dysfunction (19.0% vs. 39.3%, *P* = .017) and postoperative pneumonia (8.6% vs. 23.2%, *P* = .033) than the control group.

**Conclusion:**

Utilizing PPV as a monitoring index for GDFT can improve short-term prognosis in elderly patients undergoing laparoscopic radical resection of colorectal cancer.

**Registration number:**

ChiCTR2300067361; date of registration: January 5, 2023.

**Supplementary Information:**

The online version contains supplementary material available at 10.1007/s00384-024-04606-x.

## Introduction

By optimizing intraoperative hemodynamic parameters, goal-directed fluid therapy (GDFT) can maximize oxygen delivery to tissues and organs in patients undergoing abdominal surgery while preventing insufficient or excessive fluid infusion [1–3]. Multiple studies have demonstrated that GDFT provides the greatest benefit for high-risk abdominal surgery patients [4–6]. Therefore, for elderly patients with underlying diseases and organ dysfunction, GDFT can improve patient outcomes, reduce hospital stay duration, and decrease perioperative complications and readmission rates [7].

Traditional monitoring methods of GDFT mainly rely on transesophageal Doppler ultrasound and pulmonary artery catheterization, which accurately reflect cardiac output (CO) but are limited by their invasiveness and high cost [1, 8]. Currently, pulse pressure variation (PPV), a minimally invasive and accurate dynamic indicator for evaluating fluid responsiveness, is recognized by clinicians. Several studies have shown that PPV can guide GDFT effectively and enhance patient prognosis [1, 3, 9, 10].

Currently, laparoscopic radical resection of colorectal cancer is the preferred surgical approach for achieving enhanced recovery after surgery (ERAS) [11]. However, there is conflicting evidence regarding the application of PPV in laparoscopic surgery. The traditional belief suggests that pneumoperitoneum establishment may influence changes in stroke volume (SV) during respiration, affecting PPV measurement accuracy [12]. Nevertheless, it has been demonstrated that PPV can reliably assess fluid responsiveness during laparoscopic surgery when appropriate pneumoperitoneum pressures are maintained [13, 14]. To date, no relevant studies have investigated whether implementing PPV in laparoscopic radical resection of colorectal cancer can enhance rapid recovery after surgery. In this pragmatic study, we hypothesize that GDFT guided by PPV could optimize intraoperative hemodynamic management and reduce postoperative complications among elderly patients undergoing laparoscopic radical resection of colorectal cancer.

## Materials and methods

### Study design and population

This prospective randomized controlled trial (RCT) was conducted at the First Affiliated Hospital of Chongqing Medical University, China, as a single-center study. The Ethical approval for this study (2022-231) was provided by the Ethical Committee of the First Affiliated Hospital of Chongqing Medical University (Qing Yan) on 12 September 2022 and was registered in the Chinese Clinical Trial Registry (ChiCTR2300067361). Written informed consent was obtained from all eligible participants or their legal representatives.

Inclusion criteria: (1) age between 60 and 90 years; (2) American Society of Anesthesiologists (ASA) physical status score II-III; (3) patients undergoing elective laparoscopic radical resection of colorectal cancer. Exclusion criteria: (1) emergency surgical cases; (2) unplanned reoperations; (3) contraindications for arterial catheterization; (4) pre-existing arrhythmia; (5) congestive heart failure; (6) hepatic and renal insufficiency (creatinine, liver enzymes > 50% of normal value).

### Randomization and blinding

Participants were assigned randomly, in a 1:1 ratio, to either PPV guided GDFT or conventional fluid therapy using a random number generated by Microsoft Excel. Randomization was performed by an assistant who had no involvement in the study and the assignment was stored in sealed envelopes with serial numbers. The anesthesiologist was responsible for preoperative and intraoperative management as well as administering the fluid protocol. Only the performing anesthesiologist was aware of each patient’s assignment, while the postoperative follow-up team remained unaware of both randomization and overall intraoperative management. Data collected by both groups of personnel were recorded on specific data collection forms and subsequently transferred to two separate databases — one containing preoperative and intraoperative data, and another containing postoperative data. The combination of these two databases only occurred at the end of the study period. Independent statisticians conducted data assessment and analysis.

### Intervention and intraoperative management

The anesthesia management was standardized by the protocols of our department, and the entire procedure was performed by a single anesthesiologist. Blood pressure (BP), electrocardiogram (ECG), oxygen saturation (SpO2), and bispectral index (BIS) were routinely monitored upon patients’ arrival in the operating room. Radial artery puncture and catheterization were conducted before anesthesia administration, followed by monitoring of arterial blood pressure (ABP) and PPV. Anesthesia induction included midazolam at a dose of 0.05 mg/kg, propofol at 2 mg/kg, sufentanil at 0.5 µg/kg, and vecuronium at 0.15 mg/kg. Maintenance of anesthesia involved propofol infusion along with remifentanil administration, while sevoflurane inhalation maintained the minimum alveolar concentration (MAC) between 0.7 and 1. BIS values were maintained within the range of 40–50 throughout the procedure. Ventilator settings comprised tidal volume set at 8–10 ml/kg PBW, inspiratory-to-expiratory ratio (I/E) set as 1:2, respiratory rate (RR) adjusted to maintain end-tidal carbon dioxide partial pressure between 35 and 40 mmHg, while nasopharyngeal temperature was kept above 36 °C using heated liquid and warm fan devices. Additionally, intraoperative pneumoperitoneum pressure was adjusted between 10 and 12 mmHg based on surgical requirements and patient condition.

#### Fluid management

##### Control group

Solid food was allowed up to 8 h before surgery, while clear fluids were permitted up to 4 h prior. Rehydration was administered after admission based on physiological requirements, compensating for missed fasting volume, anesthesia volume expansion, and blood loss. The physiological requirements and missed fasting volume were determined using the 4-2-1 rehydration principle [7]. The fluid infusion volume in the first hour was calculated as follows: fasting loss /2 + physiological requirement + anesthesia expansion volume. The fluid infusion volumes in the second and third hours were determined by fasting loss /4 + physiological requirement + additional loss. The blood volume loss caused by bleeding was adjusted based on the actual amount of blood lost. The physiological requirements, the deficit in fasting, and the expansion of anesthesia volume were supplemented with compound electrolytes, and the bleeding amount was supplemented with hydroxyethyl starch.

##### PPV group

**Fig. 1:**
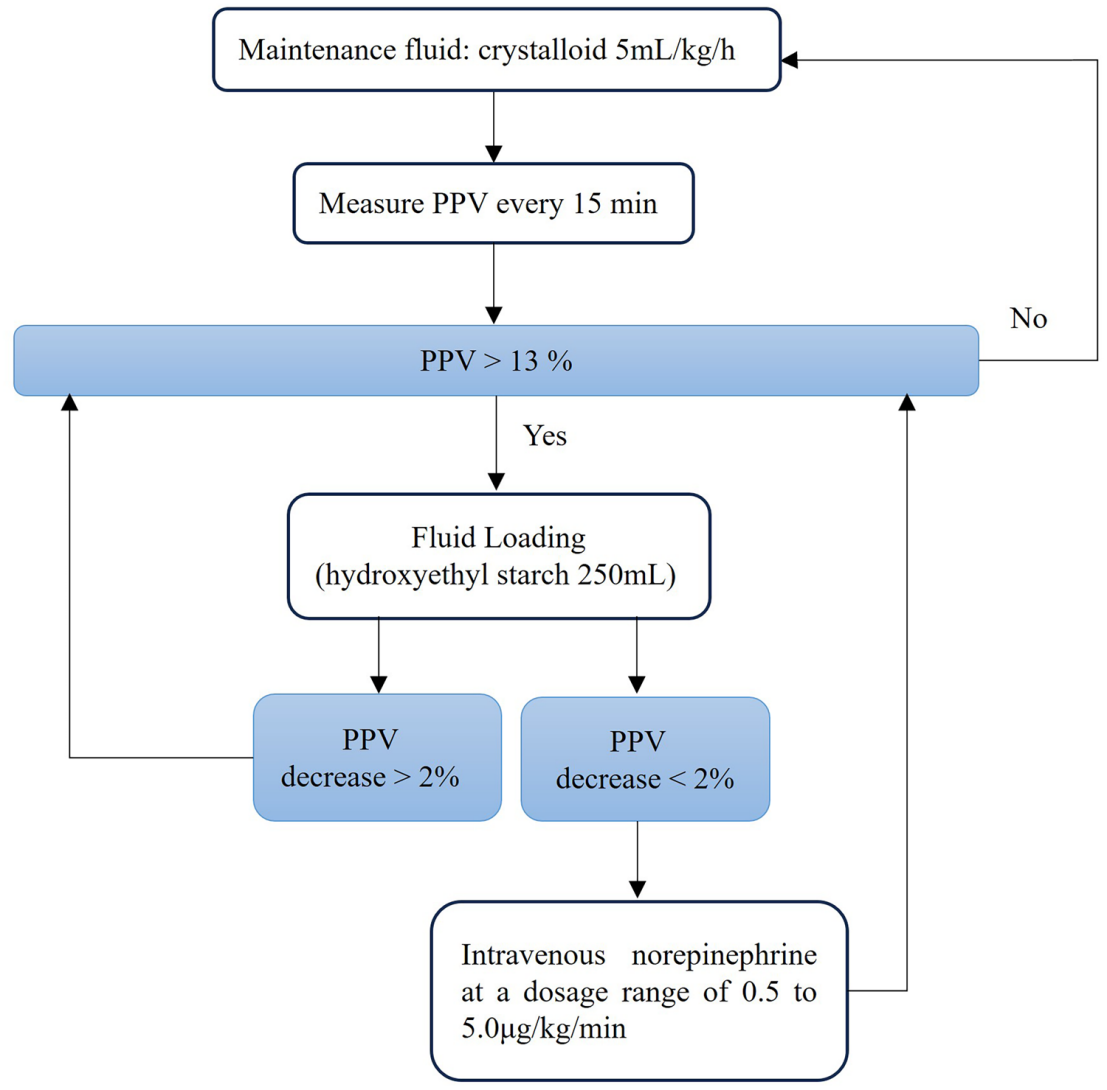
The administration of intraoperative fluids in the PPV group

Solid food was allowed up to 8 h before surgery, while clear fluids were permitted up to 4 h prior. a baseline fluid infusion volume of 5 ml/kg/h was administered upon entering the operating room. Fluid infusion was guided by PPV measurements with evaluations conducted every 15 min. No treatment was given when measured PPV ≤ 13%. If PPV > 13%, a rapid infusion of 250 ml hydroxyethyl starch took place within 10 min. After infusion, a reevaluation of PPV occurred. If there was a significant change in PPV (a decrease greater than 2% from baseline), the infusion continued until achieving the aforementioned goals. If there was not a significant change in PPV (a decrease of less than 2% from baseline), intravenous norepinephrine at a dosage range of 0.5 to 5.0 µg/kg/min would be considered until reaching this goal (Fig. 1).

#### Vital signs management

The dosage of vasoactive drugs was adjusted based on the patient’s hemodynamics, with norepinephrine, phenylephrine, and ephedrine selected according to the patient’s condition. The mean arterial pressure (MAP) was maintained at 20% of the baseline blood pressure, heart rate (HR) between 55 and 100 beats per minute, and urine output greater than 0.5 ml/kg*h. Red blood cells were transfused when hemoglobin (HB) levels dropped below 70 g/L in both groups.

### Outcome measures

The primary outcome measure was the occurrence of postoperative complications (including pneumonia, intra-abdominal infection, urinary tract infection, incisional infection, arrhythmia, heart failure, acute myocardial infarction, atelectasis, pulmonary embolism, mechanical ventilation support exceeding 24 h, urinary retention, acute renal failure, intestinal obstruction, anastomotic bleeding, and leakage as well as gastrointestinal dysfunction) within 30 days following laparoscopic radical resection for colorectal cancer in elderly patients. The diagnosis of postoperative complications was established based on a combination of strong clinical suspicion along with supporting evidence from X-ray or ultrasound imaging or laboratory tests (Table 1).

**Table 1:** Definitions of postoperative complications

Complication	Diagnostic criteria
Infection	
Pneumonia	Patients were treated with antibiotics for suspected pneumonia and had at least one of the following criteria: confirmed diagnosis via sputum culture, chest imaging examination confirmation, body temperature over 38 °C, or white blood cell count exceeding 12 × 10^9^/L.
Abdominal infection	The abdominal pain and pathogenic examination of the drainage fluid yielded positive results.
Urinary tract infections	Dysuria and positive urine pathogen tests were also noted.
Incision infection	The incision exhibited erythema, edema, increased temperature, and tenderness with purulent discharge, and the pathogen of pus was positive.
Sepsis	Positive blood cultures and at least two of the following signs: abnormal body temperature, rapid heart rate, fast breathing, or abnormal white blood cell count.
Cardiovascular complications	
Arrhythmology	ECG diagnosis of new arrhythmia requiring at least a pharmacologic intervention.
Heart failure	The symptoms and signs of heart failure (shortness of breath, rales, jugular vein distention, and edema, the third heart sound, imaging, and ultrasound signs: cardiomegaly, interstitial or alveolar edema), brain natriuretic peptide (BNP) value ≥ 35 pg/ml.
Acute myocardial infarction	The presence of new Q wave changes or persistent ST-T segment changes accompanied by abnormal troponin values was observed.
Respiratory complications	
Atelectasis of lung	Diagnosis by imaging examination
Pulmonary embolism	Diagnosis by imaging examination
Ventilator support > 24 h or ventilator weaning failure	Diagnosis by the clinical manifestations.
Kidney-related complications	
Urinary retention	The bladder is filled with urine, leading to an abnormal retention of over 100 ml.
Acute kidney injury	Any two increases in serum creatinine > 26.5 umoL/L within 48 h after surgery, or an increase in serum creatinine > 1.5 times the baseline value within 7 days after surgery (baseline value was the lowest creatinine within 30 days before surgery)
Gastrointestinal complications	
Intestinal obstruction	Abdominal pain, abdominal distension, vomiting, cessation of exhaust and defecation, and imaging examination were confirmed
Anastomotic bleeding	After the operation, the patient had recurrent bloody stool or was confirmed by endoscopy or surgery
Anastomotic leak	The presence of abdominal pain, abdominal distention, purulent secretions drainage, and confirmed imaging findings was observed.
Gastrointestinal dysfunction	The diagnosis was made according to the I-FEED scoring criteria

Other outcome measures included the Clavien-Dindo complication score at 30 days post-surgery [15, 16] and the I-FEED score for gastrointestinal dysfunction at 30 days post-surgery [11, 17]. Additionally, various physiological parameters such as HR, MAP, potential of hydrogen (PH), and lactic (Lac) values were assessed at significant intraoperative time points. Intraoperative, postoperative, and perioperative total opioid consumption was measured in morphine equivalents [morphine (iv)1 mg = remifentanil (iv)10 µg = sufentanil (iv)1 µg = tramadol (iv)10 mg] [18]. Postoperative recovery markers evaluated were extubation time, initiation of oral intake, first exhaust time, first defecation time, initial mobilization after surgery, and length of hospital stay (LOS). (Discharge decisions were guided by standardized criteria: patients should be considered ready for hospital discharge when there is tolerance of oral intake, recovery of lower gastrointestinal function, adequate pain control with oral analgesia, ability to mobilize and self-care, and no evidence of complications or untreated medical problems [19]).

### Sample size and statistical analysis

The study was designed as an RCT. The PPV group received GDFT guided by PPV, while the control group received conventional fluid therapy. The primary outcome measure assessed in this study was the rate of complications within 30 days. Based on a previous study by Mayer et al., which examined high-risk patients undergoing elective major abdominal surgery, the complication rate was 0.2 in the experimental group and 0.5 in the control group [20]. With a two-sided *α* = 0.05, a power of 1-β set at 0.9, and a sample size ratio of 1:1 between the PPV and control groups, we calculated that each group would require 48 participants using R language analysis software. Considering 20% of cases of loss to follow-up or refusal to participate, we aimed for a final minimum sample size of 60 participants in both groups, resulting in a total sample size of 120 cases.

An independent statistician utilized SPSS for Windows software (version 26.0; SPSS, Chicago, Illinois, USA) to conduct the statistical analysis. The primary outcome measures were evaluated using either the chi-square test or Fisher’s exact test. The normal distribution of the secondary outcome data was assessed using the Kolmogorov–Smirnov test. For normally distributed data, an independent sample *t*-test was employed for analysis, and variables were expressed as mean ± standard deviation (SD). Non-normally distributed data were compared using the Mann–Whitney *U* test and presented as the median and interquartile range (IQR). Count data were analyzed using either the chi-square test or Fisher’s exact test, with further pairwise comparisons conducted utilizing the Bonferroni method where appropriate. Two-factor repeated measures analysis of variance was used to compare HR, MAP, PH, and Lac.

## Results

### Study population

**Fig. 2:**
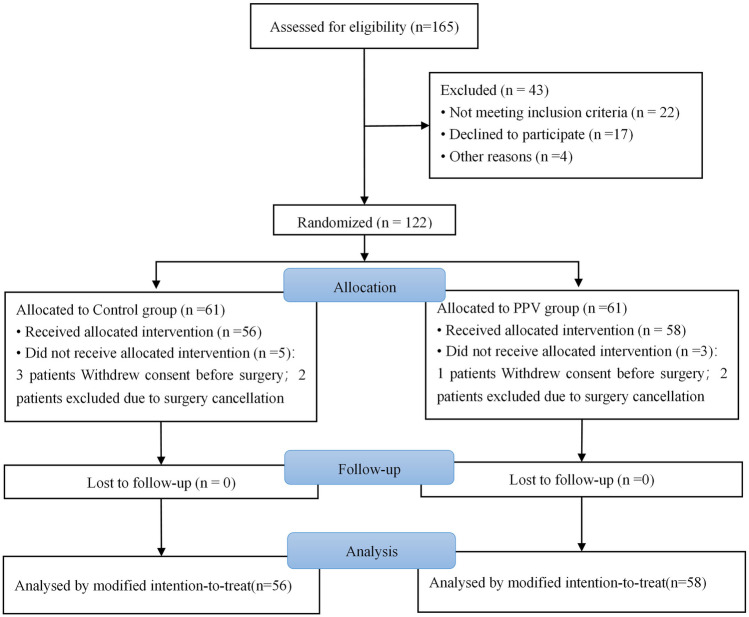
Enrollment, randomization, and follow-up

From January 2023 to November 2023, a total of 165 patients were assessed for eligibility, out of which 122 subjects were randomly assigned. Specifically, 61 patients were allocated to the PPV group and another 61 patients to the control group. In the control group, five patients did not receive the assigned intervention due to three cases of consent withdrawal before the procedure initiation and two cases of canceled procedures. Similarly, in the PPV group, three patients did not receive their assigned intervention due to one case of consent withdrawal before surgery and two cases of canceled surgeries. Consequently, a modified intention-to-treat analysis was conducted on the remaining cohort consisting of 114 patients (58 in the PPV group and 56 in the control group) (Fig. 2). Notably, both groups exhibited balanced demographic and perioperative characteristics without any significant differences observed in median operative time, laparoscopic time, or duration of anesthesia as presented in Table 2.

**Table 2:** Basic characteristics of the study subjects

	PPV group (*n* = 58)	Control group (*n* = 56)	*P*-value
Age (years)	70.2 ± 7.0	70.0 ± 7.5	0.778
BMI(kg/m2)	22.7 ± 3.1	23.0 ± 2.7	0.677
Sex			0.373
Male	38 (65.5)	41 (73.2)	
Female	20 (34.5)	15 (26.8)	
ASA physical status score			0.679
II	25 (43.1)	22 (39.3)	
III	33 (56.9)	34 (60.7)	
Coexisting medical condition			
Hypertension	18 (31.0)	18 (32.1)	0.899
Diabetes	12 (20.7)	12 (21.4)	0.923
Chronic heart disease	8 (13.8)	5 (8.9)	0.414
Chronic lung disease	5 (8.6)	8 (14.3)	0.341
Cerebrovascular diseases	3 (5.2)	6 (10.7)	0.453
Site of surgery			0.854
Colon	30 (51.7)	28 (48.3)	
Rectum	28 (50.0)	28 (50.0)	
Duration of laparoscopy (min)	129.8 ± 45.1	127.2 ± 43.4	0.761
Duration of surgery (min)	188.0 ± 54.0	189.6 ± 54.1	0.873
Duration of anesthesia (min)	229.3 ± 56.3	227.8 ± 57.0	0.891

Data are presented as mean ± SD

*BMI* body mass index, *ASA* American Society of Anesthesiologists

### Perioperative management

The crystalloid, colloid, and total fluid intake in the PPV group were significantly lower compared to the control group (*P* < 0.05). Moreover, a significantly lower number of patients in the PPV group required phenylephrine administration compared to the control group (*P* < 0.001). Additionally, although not reaching statistical significance (*P* = 0.056), there was a trend towards fewer cases requiring norepinephrine in the PPV group. No statistically significant differences were observed between the two groups regarding blood loss, urine output, number of blood transfusions, and use of ephedrine (Table 3).

**Table 3:** Intraoperative volume management and vasoactive drug use

	PPV group (*n* = 58)	Control group (*n* = 56)	*P*-value
Crystalloid infusion volume (ml)	1100 (937,1300)	1600 (1225,1700)	< 0.001
Colloid infusion volume (ml)	500 (250,500)	500 (500,500)	< 0.001
Total infusion volume (ml)	1450 (1237,1700)	2100 (1800,2375)	< 0.001
Blood loss (ml)	50 (20,100)	50 (50,100)	0.287
Urine volume (ml)	300 (150,562)	400 (162,600)	0.393
Number of blood transfusions	3 (5.2)	4 (7.1)	0.962
Number of patients using norepinephrine	15 (25.9)	24 (42.9)	0.056
Number of patients using phenylephrine	12 (20.7)	24 (42.9)	0.011
Number of patients using ephedrine	12 (24.0)	19 (35.2)	0.112

Data are presented as number (percentage) or median (interquartile range)

**Fig. 3:**
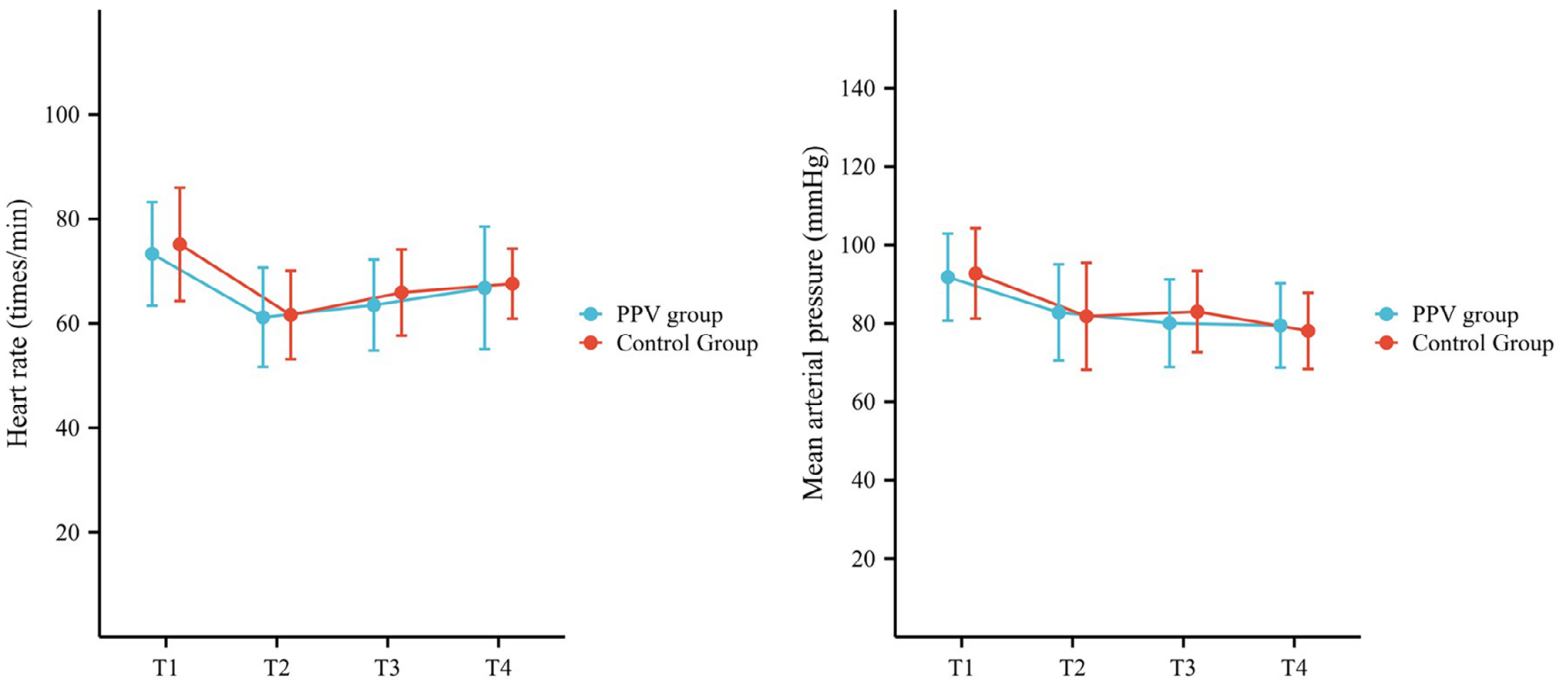
The intraoperative heart rate (HR) and mean arterial pressure (MAP). T1, before the administration of anesthesia; T2, at the initiation of surgery; T3, 30 min after the initiation of surgery; T4, at the end of surgery

The HR and MAP at T2-T4 in both groups exhibited a significant decrease compared to T1 (*P* < 0.01). There was no statistically significant difference in HR and MAP between the two groups at T1-T4. The changes in HR and MAP are illustrated in Fig. 3, with precise values provided in Appendix 1.

**Fig. 4:**
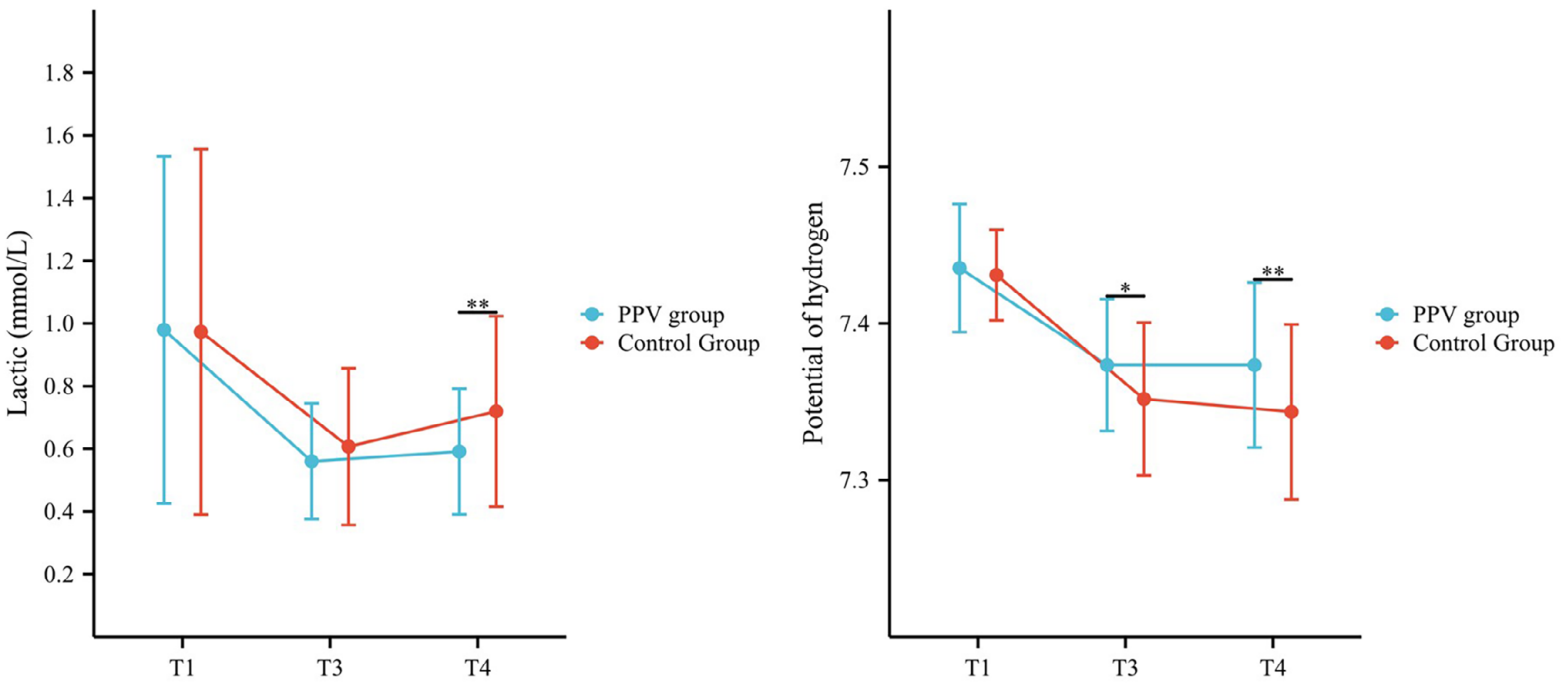
The intraoperative Lac and pH values. T1, before the administration of anesthesia; T3, 30 min after the initiation of surgery; T4, at the end of surgery. Note: *The difference between the control and PPV groups was statistically significant (*P* < 0.05). **The difference between the control group the and PPV group was highly statistically significant (*P* < 0.01)

At T1 and T3, there was no statistically significant difference in Lac levels between the PPV group and the control group. However, at T4, the Lac level in the PPV group was significantly lower than that in the control group (0.59 ± 0.20 mmol/L vs. 0.72 ± 0.30 mmol/L, *P* = 0.009). Similarly, there was no significant difference in pH between the PPV group and the control group at T1; however, at T3 and T4, the pH value in the PPV group was significantly higher than that in the control group (7.37 ± 0.04 vs. 7.35 ± 0.05, *P* = 0.012; 7.37 ± 0 0.05 vs 0.7 0.34 ± 0.06, *P* = 0.004). Lac and PH changes are shown in Fig. 4, with the exact values given in the Supplementary file, Appendix 1.

The opioid consumption (expressed as morphine equivalents) of the two groups is presented in Table 4. There were no statistically significant differences observed in terms of intraoperative morphine equivalents, postoperative morphine equivalents, and perioperative total morphine equivalents between the two groups.

**Table 4:** Intraoperative, postoperative, and perioperative total opioid consumption (expressed as morphine equivalents)

	PPV group (*n* = 58)	Control group (*n* = 56)	*P*-value
Intraoperative morphine equivalents (mg)	180.1 ± 36.1	189.6 ± 29.2	0.125
Postoperative morphine equivalents (mg)	40.4 ± 19.2	41.8 ± 19.8	0.697
Total morphine equivalents (mg)	220.5 ± 41.4	231.5 ± 34.4	0.129

Data are presented as mean ± SD

### Primary and secondary outcomes

#### Primary outcomes

Complications occurred in 19 out of 58 patients (32.8%) in the PPV group compared to 32 out of 56 patients (57.1%) in the Control group (*P* = 0.009). The incidence of gastrointestinal dysfunction was significantly lower in the PPV group compared to the control group (11 vs. 22 cases, *P* = .017), as well as pneumonia (5 vs. 13 cases, *P* = 0.033) (Table 5).

**Table 5:** The occurrence of complications within 30 days

Complications	PPV group (*n* = 58)	Control group (*n* = 56)	*P*-value
Any complications occurred within 30 days	19 (32.8)	32 (57.1)	0.009
Infection			
Pneumonia	5 (8.6)	13 (23.2)	0.033
Abdominal infection	5 (8.6)	4 (7.1)	1.000
Urinary tract infections	1 (1.7)	2 (3.6)	0.975
Incision infection	3 (5.2)	3 (5.4)	1.000
Sepsis	1 (1.7)	3 (5.4)	0.586
Cardiovascular complications			
Arrhythmology	1 (1.7)	3 (5.4)	0.586
Heart failure	3 (5.2)	5 (8.9)	0.433
Acute myocardial infarction	0	0	
Respiratory complications			
Atelectasis of lung	2 (3.4)	7 (12.5)	0.149
Pulmonary embolism	0	0	
Ventilator support > 24 h or ventilator weaning failure	0	0	
Kidney-related complications			
Urinary retention	1 (1.7)	2 (3.6)	0.975
Acute kidney injury	0	0	
Gastrointestinal complications			
Intestinal obstruction	2 (3.4)	3 (5.4)	0.968
Anastomotic bleeding	1 (1.7)	0 (0.0)	1.000
Anastomotic leak	2 (3.4)	4 (7.1)	0.643
Gastrointestinal dysfunction	11 (19.0)	22 (39.3)	0.017
Unplanned admission to the ICU	1 (1.7)	2 (3.6)	0.975
Unplanned secondary surgery	0 (0.0)	2 (3.6)	0.239

Data are presented as numbers (percentages)

#### Other secondary outcome measures

**Fig. 5:**
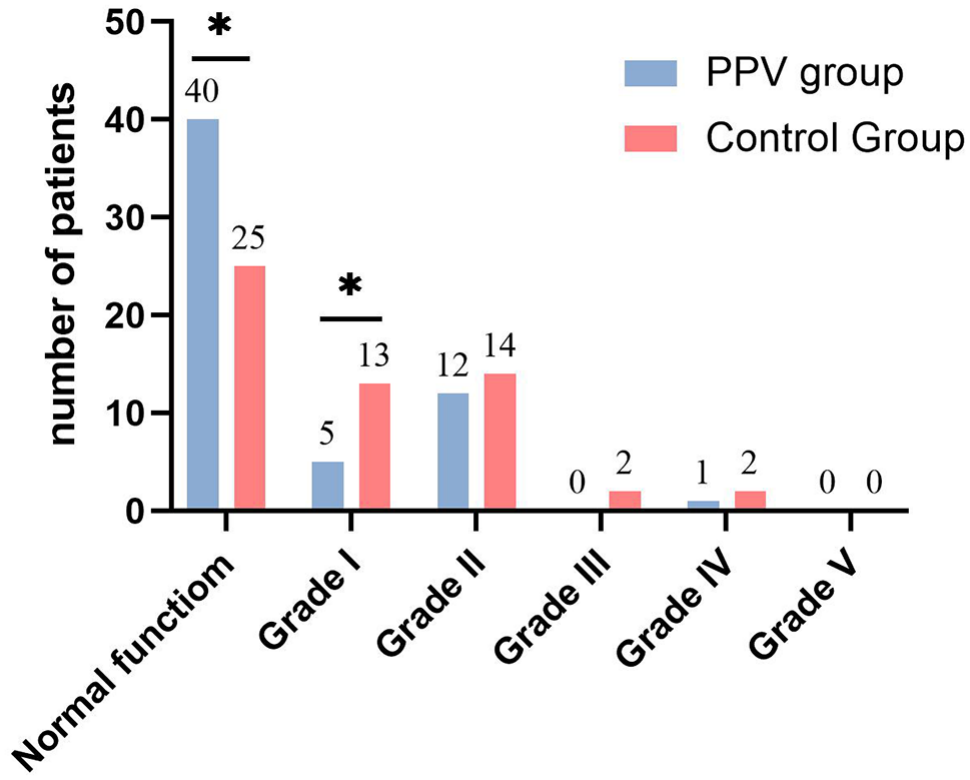
The Clavien-Dindo complication score at 30 days after surgery. Note: *The difference between the control and PPV groups was statistically significant (*P* < 0.05)

The distribution of Clavien-Dindo complication scores 30 days after surgery showed a significant difference between the PPV group and the control group (*p* = 0.032). Furthermore, based on Bonferroni analysis, there were significant differences observed in the distribution of Clavien-Dindo complication scores within the normal function subgroup (PPV group: 40 cases vs. Control group: 25 cases) and grade I (PPV group: 5 cases vs. Control group: 13 cases), while no significant difference was found in the distribution of grade II to V (Fig. 5).

**Fig. 6:**
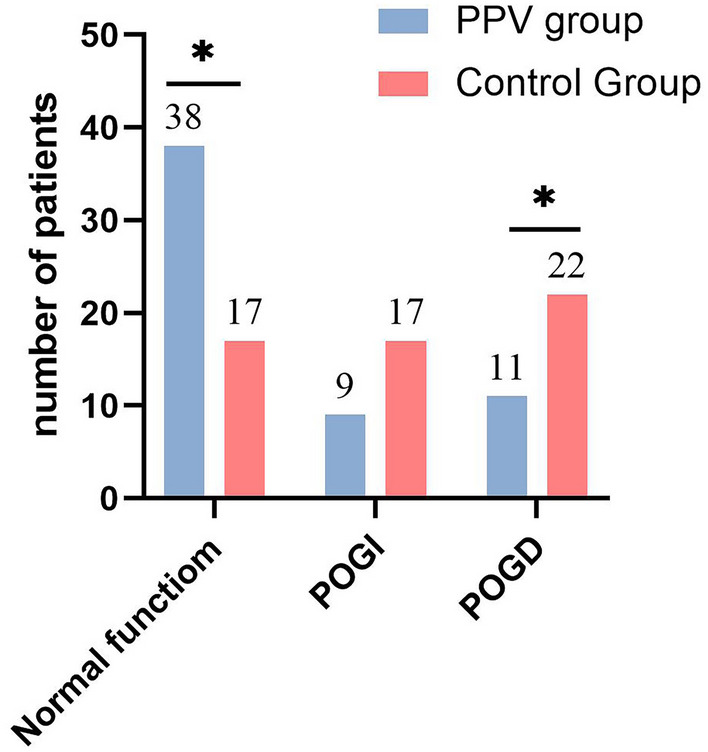
The I-FEED score for gastrointestinal dysfunction at 30 days after surgery. POGD, postoperative intestinal dysfunction; POGI, postoperative gastrointestinal intolerance Note: *The difference between the control group and the PPV group was statistically significant (*P* < 0.05)

The distribution of I-FEED scores for gastrointestinal dysfunction 30 days after surgery showed a significant difference between the PPV group and the control group (*P* = 0.001). Furthermore, applying the Bonferroni method revealed significant differences between the two groups in terms of normal function (PPV group: 38 cases vs. Control group: 17 cases) and postoperative intestinal dysfunction (POGD) (PPV group: 11 cases vs. control group: 22 cases), while no significant difference was observed in postoperative gastrointestinal intolerance (POGI) (Fig. 6).

The time to the first postoperative defecation was significantly shorter in the PPV group compared to the control group [58.5 (36.3, 79.0) h vs. 73.5 (41.8, 113.3) h, *P* = 0.040]. In the PPV group, there was a trend towards shorter durations for extubation, first food intake, and first flatus; however, statistical significance was not reached for these variables. No significant difference was observed between the two groups regarding the time to first ambulation after surgery. Furthermore, LOS in the PPV group was significantly shorter than that in the control group [11.0 (9.0, 14.0) days vs. 13.0 (11.0, 16.0) days, *P* = 0.007] (Table 6).

**Table 6:** Postoperative recovery and length of hospital stay (LOS) of patients

	PPV group (*n* = 58)	Control group (*n* = 56)	*P*-value
Postoperative extubation time (min)	20 (15, 40)	25 (20, 35)	0.541
The first postoperative feeding time (h)	47.5 (21.8, 71.3)	65.0 (20.5, 87.5)	0.548
The first postoperative exhaust time (h)	36.0 (20.0, 54.3)	39.0 (20.0, 68.0)	0.470
The first postoperative defecation time (h)	58.5 (36.3, 79.0)	73.5 (41.8, 113.3)	0.040
The first postoperative ambulation time (h)	48.0 (25.5, 69.0)	46.5 (23.0, 70.3)	0.858
LOS (D)	11.0 (9.0, 14.0)	13.0 (11.0, 16.0)	0.007

Data are presented as median (interquartile range)

*LOS* length of hospital stay

## Discussion

The key findings of this prospective randomized controlled trial (RCT) demonstrated the efficacy of PPV in GDFT, even during laparoscopic surgery. PPV guided GDFT significantly reduced the incidence of complications and shortened both gastrointestinal recovery time and LOS in patients undergoing laparoscopic radical resection for colorectal cancer.

Due to the impact of pneumoperitoneum on respiratory function, the current application of PPV in laparoscopic surgery remains a subject of controversy [12]. Recent studies have demonstrated that PPV can effectively assess volume responsiveness under appropriate pneumoperitoneum conditions [13]. It has been established that GDFT can optimize early CO, enhance organ oxygen delivery, and contribute to reducing postoperative complications. Its significance is particularly pronounced in high-risk populations such as elderly patients compared to the general population [5, 6]. Therefore, this study selected elderly patients who did not receive perioperative ERAS protocol to investigate whether PPV guided GDFT could improve the prognosis of laparoscopic surgery.

Previous studies have demonstrated that excessive fluid administration can lead to an increase in postoperative complications [21, 22]. Our study revealed that compared with conventional fluid therapy, the PPV group received significantly less fluid volume without a significant difference in urine output when compared with the control group. Furthermore, at the end of surgery, Lac levels were lower and pH values were higher in the PPV group than in the control group, indicating that fluid infusion volume in the PPV group adequately met tissue perfusion needs while excessive fluid administration in the control group may result in volume overload and hinder tissue oxygenation.

Tissue edema resulting from fluid overload can impair the functions of vital organs such as the heart, lungs, and gastrointestinal tract [7, 23–27]. Previous studies have demonstrated that intestinal edema caused by excessive fluid administration may hinder gastrointestinal transit and reduce intestinal contractile activity, leading to gastrointestinal dysfunction[28, 29]. Our study assessed the incidence of postoperative gastrointestinal dysfunction using the I-FEED score and found a significant reduction in its occurrence in the PPV group. Further evaluation of postoperative gastrointestinal function distribution revealed a significantly higher number of patients with completely normal gastrointestinal function in the PPV group than in the control group, which was consistent with the previous study [17, 30]. Additionally, we noted a shorter first postoperative defecation time and trends towards shorter times for extubation, food intake, exhaust time, and defecation time among patients receiving GDFT. The study also observed a decrease in the occurrence of postoperative pneumonia, potentially attributed to the capacity of GDFT to enhance systemic oxygenation, prevent ischemia-reperfusion injury, and mitigate pulmonary edema [31].

Inadequate fluid perfusion can have negative effects on tissue perfusion and tissue oxygenation [32, 33]. Studies conducted by Benes et al. [4] and Chytra et al. [34] have demonstrated that GDFT can decrease Lac levels in surgical patients. However, both of them administered more fluids to the experimental groups compared to the control group, whereas our study found that the PPV group received less fluid due to protocol requirements. Our findings indicate that PPV guided GDFT effectively optimizes perfusion even with reduced fluid administration, thereby preventing compromised tissue oxygenation caused by inadequate or excessive fluids. Additionally, it has been reported that the change in Lac is slow and lacks specificity. Therefore, in future studies, SvO2/ScvO2, PCO2 gap, and PCO2 gap over the arteriovenous difference in oxygen content, an estimate of the respiratory quotient and gastric mucosal pH value could be additionally incorporated for a more comprehensive assessment [35–37].

In terms of intraoperative management, both groups exhibited a decrease in MAP and HR following induction, which can be attributed to the vasodilatory effects and myocardial depression induced by anesthetic drugs. Our study demonstrated that GDFT based on PPV could effectively reduce the utilization of vasoactive drugs, aligning with the findings reported by Benes et al. [38] and Goepfert et al. [39]. This may be attributed to PPV’s ability to promptly identify inadequate intravascular volume and guide appropriate fluid administration to enhance CO thereby minimizing reliance on excessive vasoactive drug usage. Furthermore, our study revealed that the PPV group required fewer vasoactive drugs compared to the control group, consequently reducing their impact on tissue and organ perfusion. This observation suggests that decreased reliance on vasoactive drugs might serve as a potential factor contributing to reduced complications within the PPV group.

This study has certain limitations. Firstly, the PPV group in this study solely relied on PPV for guiding volume therapy without monitoring cardiac index (CI) and other indicators, which limited the accurate application of positive inotropic drugs in conjunction with volume therapy. However, recent studies by Benes et al. [38], Cannesson et al. [5], and others have demonstrated that even without CO and SV monitoring, PPV guided GDFT can still yield positive outcomes. The utilization of PPV alone for guiding volume therapy circumvents the need for expensive instruments, reduces monitoring costs, and facilitates the implementation of volume monitoring programs. Secondly, the control group in our study was assigned to a liberal fluid regimen, thus, we did not compare the GDFT with the restrictive fluid regimen. Previous studies have demonstrated that restrictive fluid regimens can mitigate complications associated with fluid overload but may result in renal injury and tissue hypoperfusion [25]. In future investigations, a comparative analysis of the effects of these three regimens on intraoperative management and patient prognosis could be conducted. Additionally, our findings cannot be directly extrapolated to all patients undergoing abdominal surgery due to the limited scope of our study population consisting solely of elderly individuals undergoing laparoscopic radical resection of colorectal cancer while maintaining an intraoperative pneumoperitoneum pressure between 10 and 12 mmHg. Finally, this study had a small sample size, restricted observation time, and lacked long-term survival follow-up; hence, further research is warranted to validate the enduring benefits of PPV guided GDFT in these patients.

## Conclusion

This study provides evidence supporting the utilization of PPV as a monitoring indicator for GDFT to facilitate precise fluid management in elderly patients undergoing laparoscopic radical resection of colorectal cancer, thereby optimizing intraoperative hemodynamic control, minimizing complications, and enhancing short-term outcomes.

## Supplementary Information

Below is the link to the electronic supplementary material.

Supplementary file1 (DOCX 17 KB)

## Data Availability

Data can be requested by the corresponding author.
