# Supplementary material for: Pulse pressure variation guided goal-direct fluid therapy decreases postoperative complications in elderly patients undergoing laparoscopic radical resection of colorectal cancer: a randomized controlled trial
Source: Int J Colorectal Dis. 2024 Mar 4;39(1):33. doi: 10.1007/s00384-024-04606-x (PMC10912221; doi:10.1007/s00384-024-04606-x)
Supplement: Supplementary file 1 — Supplementary file1 (DOCX 17 KB) [file 384_2024_4606_MOESM1_ESM.docx]

**Appendix 1.** **Hemodynamic parameters intraoperative**

|  | **Group** | **T1** | **T2** | **T3** | **T4** |
| --- | --- | --- | --- | --- | --- |
| **HR**  [bpm] | PPV Group | 73.3±9.9 | 61.2±9.5 | 63.5±8.7 | 66.8±11.7 |
|  | Control Group | 75.1±10.9 | 61.6±8.5 | 65.9±8.3 | 67.6±6.7 |
| **MAP**  [mmHg] | PPV Group | 91.8±11.1 | 82.8±12.2 | 80.1±11.2 | 79.5±10.8 |
|  | Control Group | 92.7±11.5 | 81.8±13.7 | 83.0±10.4 | 78.0±9.7 |
| **Lac**  [mmol/L] | PPV Group | 0.98±0.55 | - | 0.56±0.18 | 0.59±0.20 |
|  | Control Group | 0.97±0.58 | - | 0.61±0.25 | 0.72±0.30 |
| **PH** | PPV Group | 7.44±0.04 | - | 7.37±0.04 | 7.37±0.05 |
|  | Control Group | 7.43±0.03 | - | 7.35±0.05 | 7.34±0.06 |

HR, Heart rate; MAP, mean arterial pressure; Lac, lactic; PH, potential of hydrogen.
